# Paternal impact on the developmental programming of sexual dimorphism

**DOI:** 10.3389/fcell.2024.1520783

**Published:** 2024-12-06

**Authors:** Shefa’ M. Aljabali, Shruta Pai, Raffaele Teperino

**Affiliations:** ^1^ Institute of Experimental Genetics, Helmholtz Munich GmbH, German Research Center for Environmental Health, Neuherberg, Germany; ^2^ DZD – German Center for Diabetes Research, Neuherberg, Germany

**Keywords:** sexual dimoprhism, developmental programing, paternal inheritance, epigenetics, sperm RNAs, seminal plasma, imprinting

## Abstract

Sexual dimorphism involves distinct anatomical, physiological, behavioral, and developmental differences between males and females of the same species, influenced by factors prior to conception and during early development. These sex-specific traits contribute to varied phenotypes and individual disease risks within and across generations and understanding them is essential in mammalian studies. Hormones, sex chromosomes, and imprinted genes drive this dimorphism, with over half of quantitative traits in wildtype mice showing sex-based variation. This review focuses on the impact of paternal non-genetic factors on sexual dimorphism. We synthesize current research on how paternal health before conception affects offspring phenotypes in a sex-specific manner, examining mechanisms such as DNA methylation, paternally imprinted genes, sperm RNA, and seminal plasma. Additionally, we explore how paternal influences indirectly shape offspring through maternal behavior, uterine environment, and placental changes, affecting males and females differently. We propose mechanisms modulating sexual dimorphism during development, underscoring the need for sex-specific documentation in animal studies.

## Introduction

Sexual dimorphism (SDM) refers to the phenotypic variations between males and females within the same species. The global prevalence of SDM across sexually reproducing species has been documented in a wide range of studies. Sex differences have significantly been evident in phenotypic traits, gene expression, and diseases. A broad spectrum of qualitative and quantitative phenotypic traits, ranging from behavioral to health-related, have been observed to display significant sex differences in many mammalian species, most notably in humans (Choleris et al., 2018; Karp et al., 2017; Weiss et al., 2006; Rawlik et al., 2016). In terms of gene expression, advanced technologies such as qRT-PCR, microarray as well as bulk and single-cell RNA-seq analyses, have identified a few hundred to several thousands of sexually dimorphic genes in a variety of animal and human tissues such as liver, muscles, adipose tissue, brain, *etc.* These genes are involved in a wide range of biological processes, mainly related to metabolism, endocrine and immune response among many other cell signaling pathways (Yang et al., 2006; Seo et al., 2016; Oliva et al., 2020; Khodursky et al., 2022). In the context of diseases, the influence of SDM can be apparent in various ways, including disease course, onset, expressivity, associated symptoms, prognosis, and prevalence. Since identifying and understanding sex disparities in diseases is therapeutically relevant, many diseases have been investigated in a sex-specific manner. Specifically, cardiovascular diseases (Miller, 2012), renal diseases (Neugarten and Golestaneh, 2019), neurological diseases (Zalewska et al., 2022), autoimmune diseases (McCombe et al., 2009), respiratory diseases (Reddy and Oliver, 2023), infectious diseases (Gay et al., 2021), and several types of cancers (Ma et al., 2016) in both human and animal models exhibit clear sexual dimorphism.

Given the prevalence of SDM across diverse biological and physiological aspects, a mechanistic understanding of its ontogeny is crucial to dissecting and comprehending the sex differences in complex traits and diseases. While still not entirely understood, the main underlying mechanisms include sex chromosomes and sex hormones, but also involve epigenetic modifications and sex specificities during developmental programming. Extensive studies have unequivocally shown that gonadal sex hormones (estrogens, progestins, and androgens) are primary mediators in a wide array of phenotypes displaying SDM. The sexually dimorphic effects of sex hormones can be either organizational (permanent) or activational (reversible). However, the emergence of sex differences before the development of gonads (Lowe et al., 2015; Werner et al., 2017) suggests that genetic and epigenetic factors are involved in SDM. Sex chromosome effects refer to the differential action of genes present on the sex chromosomes in female (XX) and male (YX) cells. Sex chromosome effects can be mainly attributed to the presence of the Y chromosome and the role of X chromosome dosage (Snell and Turner, 2018). Using sex-chromosome-modified mouse models such as XY* and Four Core Genotypes (FCG) has effectively dissected whether a phenotypic sex difference is influenced by sex chromosomes, gonadal sex hormones, or interactions of the two (Burgoyne and Arnold, 2016; Blencowe et al., 2022).

**FIGURE 1:**
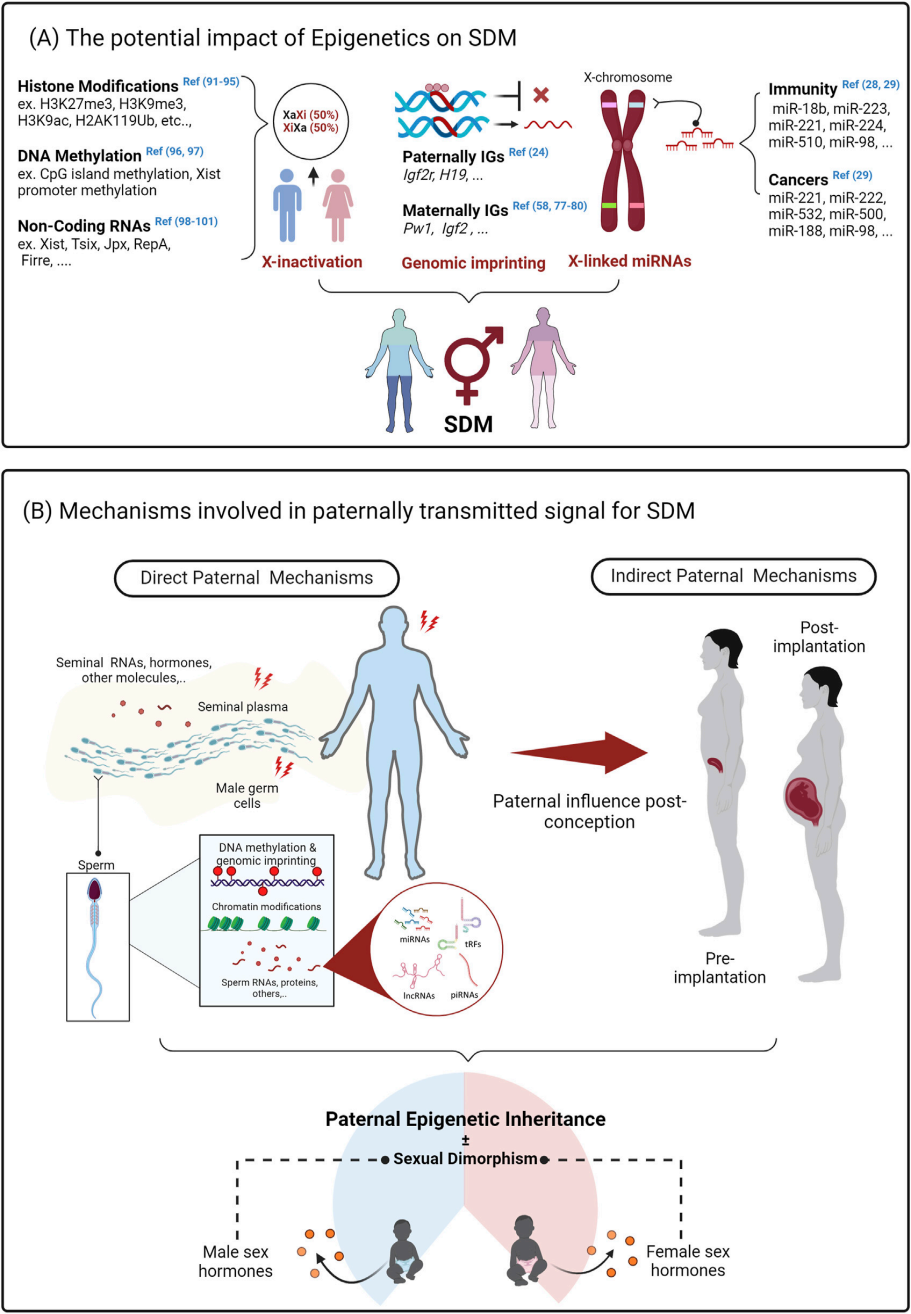
Graphical illustration of how epigenetics may impact sexual dimorphism and how paternal inheritance mechanisms can affect offspring phenotypes in a sex-specific manner. **(A)** highlights the main epigenetic mechanisms that potentially affect sexual dimorphism (SDM), including X-chromosome inactivation (XCI), genomic imprinting, and X-linked miRNAs. Male and female offspring can be potentially affected differently by fathers in both direct and indirect ways as shown in **(B)**. For instance, direct mechanisms involve sperm-borne factors such as DNA methylation and imprinting, chromatin modifications, small non-coding RNAs, and other factors in the seminal plasma. Indirect ways include paternal influences on maternal behavior, the uterine environment, and placental alterations. Although human figures (male, female, and infant) are used to illustrate sex-specific variance of paternal epigenetic inheritance mechanisms, these mechanisms are primarily inferred from animal models, which still require confirmation in human studies. Abbreviations: H3K27me3 – Histone H3 lysine 27 trimethylation, H3K9me3 – Histone H3 lysine 9 trimethylation, H3K9ac–Histone H3 lysine 9 acetylation, H2AK11pUb–Histone H2A lysine 11 polyubiquitination, Xist–X-inactive specific transcript, Tsix–Xist antisense transcript, Jpx–Jpx RNA, RepA–Repeat-associated non-coding RNA, Firre–Functional Intergenic Repeat RNA, Igf2r–Insulin-like growth factor 2 receptor, H19 – H19 imprinted maternally expressed transcript, Pw1 – Paternally Expressed Gene 3, Igf2 – Insulin-like growth factor 2 (Chen and Zhang, 2020; Fukuda et al., 2014; Fang et al., 2019; Żylicz et al., 2019; Sharp et al., 2011; Tinker and Brown, 1998; Panning et al., 1997; Lee et al., 1999; Froberg et al., 2013; Yang et al., 2015).

The role of epigenetics in SDM has been manifested in some key processes such as X chromosome inactivation (XCI) and genomic imprinting - Figure 1A. XCI refers to the process of silencing one of the two X chromosomes through DNA methylation, histone modifications and non-coding RNAs to achieve dosage compensation and balanced gene expression (Avner and Heard, 2001). Nevertheless, some X-linked genes (both in humans and mice) escape silencing by XCI which leads to different gene expression levels between female and male cells due to the bi-allelic expression. As a result, this provides a source of sex differences in phenotypic traits and disease susceptibility. For instance, DDX3X (Dead Box Helicase 3, X-Linked), KDM6A/UTX (Lysine Demethylase 6A/UTX), MAGEC3 (Melanoma Antigen Gene Family, Member C3), CNKSR2 (Connector Enhancer of Kinase Suppressor of Ras 2), KDM5C (Lysine Demethylase 5C), ATRX (Alpha Thalassemia/Intellectual Disability Syndrome X-Linked) are tumor suppressor X-linked genes located in the non-pseudoautosomal region (PAR) which has been reported to escape silencing by XCI and thus contribute to cancer sex bias (Dunford et al., 2017). Another example that shows the implication of XCI in SDM is the abnormal expression of the lncRNA *Xist*, an X-inactive specific transcript. In particular, the inhibition of *Xist* gene expression can suppress cell proliferation, indicating that high expression of the lncRNA *Xist* might account for the sex differences in the proliferative potential of pulmonary arterial endothelial cells in women and consequently boost their susceptibility to pulmonary arterial hypertension (Qin et al., 2021).

Genomic imprinting is another potential key player in SDM. It represents the process of suppressing a subset of genes in one parent using DNA methylation, resulting in monoallelic parental-specific expression. Since some of the imprinted genes (IGs) are involved in growth, metabolism, and brain functions (Tucci et al., 2019), it is plausible that these imprinted genes play a part in the sex differences observed in these physiological processes. To provide evidence for this speculation, the imprinted-X liability threshold model suggests that a certain imprinted X-linked gene(s) (only active when inherited paternally) is protective in nature against phenotypic expression of many autism-related traits and therefore raises the threshold for females to develop autism (Skuse, 2000). In 2010, Gregg et al. revealed through their in-depth analysis that the imprinted expression of interleukin-18, a cytokine that regulates neuroinflammation, is expressed in female brains but not in male brains (Gregg et al., 2010). Another study has also shown a link between interleukin-18 gene expression and multiple sclerosis, a highly sex-specific disease with a noticeable prevalence in females (Herrera et al., 2008).

Another epigenetic mechanism that could account for sex differences is the differential expression of X-linked microRNAs, which are known to regulate the post-transcriptional expression of numerous genes involved in immune response, cytokine production, apoptosis regulation, and cell lineage determination (Pinheiro et al., 2011; Hewagama, 2013). This notion is supported by the fact that the X chromosome carries a higher number of miRNA-encoding genes when compared to the autosomes and Y chromosome and several miRNA-encoding genes on the X-chromosome have been found to escape XCI (Migliore et al., 2021). In multiple studies, differential expression of X-linked miRNAs between the sexes has been reported as a possible mechanism behind sex differences in several diseases, including multiple sclerosis, cerebral ischemia, autoimmune diseases, and various malignancies (Migliore et al., 2021).

A growing body of evidence has shown that the epigenome can be affected and reprogrammed by environmental factors, resulting in changes to the expression of many phenotypic characteristics. Emerging studies have also demonstrated that these environmentally induced traits can be passed on from parents to offspring in a sex-specific manner. Traditionally, parentally induced epigenetic effects in offspring have been mainly attributed to mothers because there were no established mechanisms for transferring epigenetic factors between fathers and offspring. However, sperm- and ejaculate-mediated mechanisms of transfer have revealed that epigenetic effects via the paternal line can be highly significant (Lassi et al., 2021; Tomar et al., 2024; Comas-Armangue et al., 2022). Since paternal contributions are pleiotropic and have a sex-specific impact, understanding the mechanisms underlying paternal epigenetic inheritance could provide a beneficial strategy for understanding SDM in phenotypic traits and diseases.

Of particular interest for the scope of this review, SDM can emerge early in development due to epigenetically programmed differences, often influenced by the parental health status at conception. More recently, paternal health at conception has gained substantial relevance for its impact on offspring health and recent studies have highlighted that factors such as metabolic and circadian states may affect gene expression profiles in male and female embryos differently, contributing to observable sex-specific phenotypic differences in offspring, including susceptibility to metabolic and neuropsychiatric disorders (Comas-Armangue et al., 2022; Billah et al., 2022; Costa et al., 2018; Cambiasso et al., 2022; Solomon and Zerach, 2020; Sun et al., 2023) In this review, we will specifically focus on paternal programming of sexually dimorphic phenotypes and discuss molecular mechanisms and physiological relevance.

## Evidence of sexual dimorphism in paternal inheritance

Various studies have documented that environmental perturbations on parents result in sexually dimorphic impact on offspring. These studies range over multiple species with most documenting only maternal (Risal et al., 2021), whereas some comparing the impact of maternal and paternal effects (Hedegger et al., 2020). These comparisons also give a unique insight into a possible additive nature of the perturbation. Paternal inheritance is mediated directly via sperm borne factors like DNA methylation and imprinting, chromatin modifications as well as small non-coding RNAs, along with its nutritious soup, the seminal plasma- Figure 1B.

*Gasterosteus aculeatus*, commonly known as the three-spine stickleback offers a great model system to study evolutionary adaptations and epigenetic mechanisms (Reid et al., 2021). A study using the three-spine sticklebacks in freshwater has shown comparison of maternal and paternal stress using predation risk with sexually dimorphic impact on offspring brain gene expression (Hellmann et al., 2020a). The same study further reported risk-prone male offspring born from stressed fathers and anxious offspring (sex-independent) from stressed mothers (Hellmann et al., 2020a), while a second study expanded on the impact of paternal stress on F2 and found sex specific differences in offspring based on maternal/paternal lineage in F1 (Hellmann et al., 2020b). *Drosophila melanogaster*, another widely-used model to study epigenetics, has been used to show the sex-specific intergenerational impact of paternal stress, using protein restriction during larval development (Zeender et al., 2023). In particular, daughters of affected fathers showed enhanced fecundity when their diet aligned with that of the fathers compared to controls. Whereas the sons showed the same abundance of offspring but faster mating (Zeender et al., 2023).

Studies in rats show promising insight into understanding the sex-dependent variance of behavioral phenotypes. A study showed the impact of increasing doses of Morphine in male rats (during adolescence) on its offspring, and observed a sexually dimorphic impact on anxiety-like behavior in offspring (Azadi et al., 2021). Paternal self-administration of morphine also induces sexually dimorphic results in offspring, with object recognition memory deficit present in females but not male offspring (Ellis et al., 2020). A study with paternal preconception exposure to Cannabis showed male and female offspring having inverse methylation-expression relationship with their respective sex controls (Schrott et al., 2022). The study also reported cardiomegaly in offspring along with significant differences in both offspring sexes in response to addition of a washout period, but only in female offspring in the absence of a washout period (Schrott et al., 2022). Paternal preconception exposure to Nicotine has also shown sexually dimorphic outcomes, with male offspring exhibiting locomotor hyperactivity, exclusively during adolescence, and female offspring exhibiting reduced response latency (Hawkey et al., 2019).

Metabolic health of offspring is also affected by paternal impact in Rats as shown with high-protein diet that induced increased insulin sensitivity in male offspring (Gong et al., 2021). Beta cell plasticity of these paternally exposed male offspring was enhanced as well in response to high fat diet metabolic challenge (Gong et al., 2021). Studying offspring of obese fathers also showed higher susceptibility to impairment in the male offspring compared to the female offspring, when subjected to high fat diet (Sanchez-Garrido et al., 2018).

A study with rats subjected to predatory stress showed that the maternal and paternal effect was not additive, in agreement with Hellmann et al.‘s findings in three-spined sticklebacks (Hellmann et al., 2020a). The authors also made comparisons with onset of acute stress of the offspring and observed that female non-acute stressed offspring were more impacted with paternal stress in comparison to combined, whereas acutely stressed pups were more impacted with combined stress than maternal stress present alone (Azizi et al., 2019). These phenotypes offer insight into the complex nature of inheritance of SDM.

While studies with rats give more phenotypic insight into behavior, studies with mice evidently give a deeper understanding of mechanistic differences during development, and aid in hypothesizing possible mechanisms of SDM. Studies with fathers exposed to early life unexpected stress exposure with maternal separation showed that only male offspring exhibit behavior of social anxiety, although the phenotype was absent in fathers (Franklin et al., 2011). Here, depressive-like behavior of fathers was inherited to female, but not male offspring, and it could be reversed with anti-depressants. Interestingly, the second effect was inherited by grandsons through the male lineage, thus, phenotype skipping a generation. Chronic stress exposure to fathers in adulthood through social defeat has also been shown to affect the offspring, with males showing a more robust phenotype as well as exclusive increase in plasma corticosterone and decrease in Vascular endothelial growth factor (VEGF) (Dietz et al., 2011). The same study also reported that IVF did not mimic the phenotype, indicating involvement of factors other than sperm, by itself (Dietz et al., 2011). Paternal chronic stress with restraint stressor/forced swim test also shows sex specific impact on offspring anxiety-like and depression-like behavior, with males exhibiting reduction and females-an increase (Mashoodh et al., 2023). Interestingly, the changes in males have been correlated to differences in maternal investment in pregnancy. Despite these studies showing sex-specific behavioral differences, chronic paternal stress with varied stressors has been found to translate in reduced HPA axis responsiveness in the offspring, in a non-sex-specific manner, further depicting the complexities of the subject (Rodgers et al., 2013).

Exposure to various compounds has also been documented to be paternally inherited in mice. Paternal valproic acid exposure has been shown to affect behavior of both sexes similarly, but deficits in the sensorimotor gating were only observed in females (Ibi et al., 2019). Prenatal paternal exposure to alcohol has been reported to cause greater intrauterine growth restriction in males than females (Chang et al., 2019). The same study also reported metabolic dysregulation in the offspring with males having decreased glucose and insulin and females having increased insulin (Chang et al., 2019). Paternal exposure to glucocorticoids is shown to affect male offspring more than female, in the context of pro-anxiety behaviors (Short et al., 2016), whereas the same evidently affected memory retention in females, not males (Yeshurun et al., 2017). In the context of glucocorticoids, our group has recently shown disrupted levels of it in the seminal plasma as a result of circadian disruption have affect metabolic health of male offspring, and the impact on female offspring is minimal and different (e.g., Lower weight) (Lassi et al., 2021). Differences in metabolic phenotypes were also observed on paternal high fat diet treatment leading to the F2 generation, with males showing obese hyperglycemic phenotype with upregulated glycolysis and females showing lean hyperglycemic phenotype with upregulated gluconeogenesis and lipolysis (Park et al., 2018). It is likely from these observations that a different set of response cascade in males and females is in place and is dependent on the type of stress.

Finally, case studies in humans aid towards increasing the clinical relevance of all animal models. Studies with paternal exposure to organic pollutants have reported effects on sex ratio. In Michigan fish eaters, exposure to polychlorinated biphenyls have been documented to affect secondary sex ratio with increased male offspring (Karmaus et al., 2002), whereas in a Faroe island males, they found the levels of persistent organic compounds to negatively correlate with Y:X sex ratio in sperm (Kvist et al., 2014). Paternal opioid use, starting after childbirth, has been associated with significantly increased odds ratio of obesity in sons but not in daughters (Jalali et al., 2021). Paternal smoking exposure has been found to be associated with greater body mass index (BMI) in 9 year old sons (Pembrey et al., 2006). Furthermore, paternal smoking exposure is also significantly associated with childhood overweight/obesity in sons, not daughters, and the phenotype is evident with pre-conception and post-conception exposure, but not post-natal exposure (You et al., 2022). Smoking is also established to cause alterations in DNA methylation (Jenkins et al., 2017) and sperm chromatin condensation (Laqqan and Yassin, 2022), indicating an epigenetic link. Well established cohorts have also demonstrated that grandfather’s food supply is associated exclusively with grandson’s increased mortality risk ratio (Pembrey et al., 2006; Vagero et al., 2018). Various studies have also been documented for metabolic phenotype and obesity (Comas-Armangue et al., 2022), but they unfortunately don’t yet provide a clear mechanistic insight.

## Mechanisms involved in paternally transmitted signal for SDM

While numerous studies report sexual dimorphism (SDM) in phenotypes, the mechanisms governing SDM remain poorly understood. Key questions persist, such as: (a) which signals communicate parental stress versus which are passive observations, and (b) what the boundaries of programming are in terms of developmental stages or offspring age. Additionally, it remains uncertain whether these mechanisms respond uniformly to various stress types. A central debate, often referred to as the “chicken and egg problem,” questions whether SDM is developmentally programmed due to parental influence alongside genomic and epigenomic contributions, or if it emerges in adulthood through hormonal pathways and gonadal activity. Understanding how inheritance mechanisms may intersect with SDM pathways to drive significant phenotypic differences remains an intriguing area of inquiry. Currently, there are competing hypotheses suggesting both early developmental programming and postnatal activation as potential drivers of SDM.

The mechanisms of paternal inheritance mediated by both germ cell and non-germ cell components of the male reproductive tract and including DNA methylation, chromatin organization and the activity of small non-coding RNAs have been extensively reviewed (Daxinger and Whitelaw, 2012; Chen et al., 2016; Lempradl, 2020) and also summarized here in Figure 1. Global studies have identified differences such as sperm hypomethylation associated with low-protein diets (Watkins et al., 2018) and gestational arsenic exposure (Nohara et al., 2020) associated to SDM in offspring phenotypes. However, due to current methodological limitations, single-sperm chromatin and DNA methylation analyses are not yet feasible. This makes it unclear whether these differences are already encoded within sperm carrying either the X or Y chromosome. There is, however, much more evidence in support of RNA mediated inheritance.

Long term restraint stress has been shown to cause differential DNA methylation at regions, that are further inherited paternally (Zheng et al., 2021) and this has been proposed to be mediated by small non-coding RNA (sncRNA), which further highlights the interactive nature of epigenetic mechanisms. Increased abundance of several miRNAs in sperm has also been reported in the case of paternal obesity, that interestingly resulted in more pronounced transcriptomic changes in male blastocysts, compared to female (Hedegger et al., 2020). Reduced levels of certain miRNA family members in sperm from fathers with chronic social instability have been associated with elevated anxiety and defective sociability in female offspring (Champroux et al., 2024). Interestingly, restoring the miRNA member in pre-implantation embryo has shown to reduce the phenotype, which strengthens the case of RNA mediated inheritance, or at least highlights its importance for early development and late-onset phenotypes.

Spermatogenesis is a complex process and in recent years, there has been more attention towards contribution from the epididymis, either directly via epididymosomes (Chen et al., 2016), or through the environment provided for maturation. We have recently shown that the epidydimal spermatozoa are sensitive to environmental stimuli and transfer the signal through mitochondrial tRNAs (mt-tRNA) and their fragments (Tomar et al., 2024). Paternal high fat diet treatment caused a spike in these mt-tRNAs, and they were inherited at fertilization. Interestingly, despite being inherited and found in both male and female early 2-cell embryos, only male embryos were transcriptionally reprogrammed and only male offspring showed impaired metabolic homeostasis (Tomar et al., 2024). These findings support the idea that a vast proportion of sexually dimorphic phenotypes are established early in development before any possible hormonal contribution. In keeping with this, the currently available data from two-cell to pre-implantation stages has also been repurposed to study sex differences and uncovered early differences forming in two waves, both long before hormonal signaling, further supporting the theory of developmental programming (Richardson et al., 2023). Finally, we cannot rule out maternal reaction to paternal stress while defining pre-implantation programming. As mentioned before, maternal investment in the pregnancy is documented to be altered, specifically for male offspring in terms of prenatal weight gain and nursing (Mashoodh et al., 2023). Apart from behavioral reaction, mechanistic differences can also be programmed during fetal development (Watkins et al., 2020). The seminal plasma from paternal low protein diet has been shown to blunt maternal immunological responses (Watkins et al., 2018), and it has also been documented to have a sexually dimorphic impact on their vascular function, with females displaying significantly greater acetylcholine-mediated vasodilation responses to nitric oxide synthesis inhibitor, and males displaying a significant reduction (Morgan et al., 2020).

Post-implantation, placenta plays a crucial role in providing the necessary nourishment to the fetus. Fetal growth restriction has been reported as a consequence of paternal stress (Lassi et al., 2021). It is also well established that male and female placentas are different in their gene expression, miRNA profiles, as well as histopathology based on the requirement of the fetus (Gabory et al., 2013; Christians, 2022) thus being a key player in conveying stress signals in a sexually dimorphic manner. Paternal preconception stress is shown to have a divergent effect on the transcriptional profiles of placentae at E12.5, with female featuring increase in carbohydrate, lipid and amino acid metabolism, whereas males show reduction of immune-regulatory genes (Cisse et al., 2020). Another study showed that paternal high fat diet caused low placental weights for males but not females, whereas a combination with exercise indicated decrease in expression of pro-inflammatory molecule mRNAs, exclusively in female placentae (Claycombe-Larson et al., 2020). In females, this is also exclusively linked to reduced levels of sperm miRNA 193b, which indicates a sex-specific communication between father-placenta-daughter.

Parentally imprinted gene *Pw1* (Paternally Expressed Gene 1) has been shown to be involved in SDM that arises postnatally, using *Pw1* deficient mice that showed reduced masculinization of body composition in males as well as reduced testosterone at puberty and reduced growth hormones levels at early postnatal developmental points (Tanaka et al., 2022). Interestingly, this was also one of the imprinted genes found to be affected in male offspring of fathers with early life stress (Thivisol et al., 2023). *Igf2* (Insulin-like Growth Factor 2), another imprinted gene was found to be increased in offspring of fathers with glucocorticoid exposure (Short et al., 2016), as well as maternal separation induced early life stress (Thivisol et al., 2023). On the other hand, it has also been reportedly lowly expressed upon paternal stress, in adult male rat offspring hippocampus and neocortex, but not in neonatal offspring (Ordyan et al., 2022) IGF2 treatment (human recombinant) in rats during late pregnancy showed male fetuses to be more stimulated and affected, further documenting its role in SDM (White et al., 2018). These cases support the theory that SDM phenotypes are established postnatally.

Hormones also significantly influence SDM in various physiological systems including metabolism (Santos-Marcos et al., 2023; Sandovici et al., 2022) and immunity (Shepherd et al., 2020). Sex-dimorphic differences appearing early post-natal are owed to growth and sex-specific hormone related pathways and have been linked, for example, to the pulsatile nature of growth hormone release in males and continuous in females (Gabory et al., 2009). Paternal obesity is reported to reduce luteinizing hormone (LH) levels in male offspring, but not female (Sanchez-Garrido et al., 2018). It has also been recently established using paternal exposure to inorganic compounds that the metabolic phenotype in female offspring is induced by estrogen which was further confirmed with absence of phenotype using hepatic knockout of estrogen receptor α/ß (Xue et al., 2023). This sheds light on possibilities of long-term programming of stress signals in a sex-dependent manner, but doesn’t rule out an initial trigger based on epigenetic developmental programming. The above mechanisms are summarized in Table 1.

**TABLE 1:** Models and modes of paternal influence on sexual dimorphism The table summarizes documented models of paternal stimuli with their modes/mechanisms of paternal influence. We also summarize in brief, the phenotypes and observed instances of sexual dimorphism. Abbreviations: Igf2- Insulin-like Growth Factor 2, sncRNA-small non-coding RNA, tsRNA-tRNA-derived small RNAs, Pw1/Peg3- Paternally Expressed Gene 3.

Paternal stimulus	Species	Mechanism	Phenotype reported	Sexual dimorphism observed	Ref
Low protein diet	*Mus musculus*	Sperm global DNA hypomethylation within gene body regions, blunted uterine immunological response	Metabolic dysfunction, impaired vascular function	Yes	Watkins et al. (2018), Morgan et al. (2020)
High fat diet	*Mus musculus*	Elevated mt-tRNA levels in epididymal spermatozoa that are inherited	Impaired metabolic homeostasis	Yes	Tomar et al. (2024)
high-fat diet with exercise	*Mus musculus*	Altered sperm miRNAs (reduced miR-193b, increased miR-204) impact on placenta	Sex-specific placental response	Yes	Claycombe-Larson et al. (2020)
Obesity	*Mus musculus*	Differential miRNA levels in sperm causing direct impact pre-implantation (9 upregulated, 2 downregulated)	Transcriptomic changes in blastocysts	Yes	Hedegger et al. (2020)
Obesity	*Rattus norvegicus*	Reduced luteinizing hormone (LH) levels	Metabolic and reproductive impact	Yes	Sanchez-Garrido et al. (2018)
Glucocorticoid exposure	*Mus musculus*	Upregulated microRNAs (miR-98, miR-144 and miR-190b), differentially expressed imprinted gene Igf2	Anxiety and depressive phenotype	Yes	Short et al. (2016)
Glucocorticoid exposure	*Rattus norvegicus*	differentially expressed imprinted gene Igf2	Memory deficit related phenotype	Yes	Ordyan et al. (2022)
Circadian disruption	*Mus musculus*	Corticosterone in seminal plasma at conception, via fetal growth restriction	Metabolic dysfunction and altereted oscilatory transcription	Yes	Lassi et al. (2021)
Gestational arsenic exposure	*Mus musculus*	Sperm DNA hypomethylation in CpGs of retrotransposons	Increased hepatic tumor incidence	* (only males reported)	Nohara et al. (2020)
Exposure to inorganic arsenic	*Mus musculus*	Disrupted hepatic estrogen signalling	Metabolic dysfunction	Yes	Xue et al. (2023)
Paternal preconception stress (randomized seven different stressors)	*Mus musculus*	Divergent placental transcription profiles at E12.5	Metabolic and immune regulatory differences in placentae, transcriptomic differences in fetal brain	Yes	Cisse et al. (2020)
Long term restraint stress	*Mus musculus*	Differentially methylated regions in sperm, differential sncRNA in sperm (upregulated tsRNAs, downregulated miRNAs, rRNAs)	Behavioral and reproductive disorders	* (only males reported)	Zheng et al. (2021)
Chronic stress with restraint or forced-swim	*Mus musculus*	Mediated through maternal investment	Altered anxiety and depressive phenotype	Yes	Mashoodh et al. (2023)
Maternal separation stress	*Mus musculus*	Differentially expressed imprinted genes (Igf2, Pw1/Peg3)	Offspring risk taking behavior	* (only males reported)	Thivisol et al. (2023)
Chronic social instability	*Mus musculus*	Reduced levels of specific miRNA families in sperm (miR-34–449)	Elevated anxiety and sociability issues	Yes	Reddy and Oliver (2023)

## Discussion

In summary, we show evidence of the sexual dimorphic nature of paternal inheritance in various species and the current understanding of the mechanistic underpinnings behind it. However, systematically compiling the sexually dimorphic contributions of fathers towards various offspring phenotypes and complex traits has a handful of limitations underlined in the mechanistic complexities of paternal epigenetic inheritance, research design constraints, and the persistent knowledge gaps. For instance, several mechanistic routes and machineries by which paternal signals are passed on to the offspring remain enigmatic, especially throughout the early stages of development when cell signaling pathways are dynamic as well as not yet clearly defined. Moreover, male germ cell heterogeneity poses a considerable challenge for our understanding of which sperm-borne factors or signals are specifically implicated in paternal inheritance and SDM. Furthermore, investigating and studying both offspring sexes incredibly aids in unraveling the sexually dimorphic effects and ensures a clear thorough understanding of paternal impact across generations.

Our understanding of the mechanisms in humans is deeply lacking, partly owing to the ethical complications. Animal models help in driving the science in the right direction, but diseases often tend to present differently in humans compared to other animal models and it is very important to confirm these findings in humans to ascertain clinical relevance. Finally, the necessity of performing systemic phenotyping and sex-stratified analyses in upcoming studies is highly pressing since paternal signals inherited by offspring could impact a broad range of traits, other than those intended to be examined or studied. A more extensive approach that embraces various traits might uncover earlier neglected or subtle effects, reinforcing a profound understanding of the sex-specific variances of paternal epigenetic inheritance.
